# Warning Function of Frank's Sign in Pre-Existing Cardiac Disease Patients: A Case Report

**DOI:** 10.1155/2024/3766536

**Published:** 2024-07-09

**Authors:** Mingzhe Wang, Yujing Zhang, Jiang Huang, Geping Liao, Wei Qian, Yaofu Zheng, Xiaoping Peng, Jianbing Zhu

**Affiliations:** ^1^ Department of Cardiology The First Affiliated Hospital of Nanchang University Jiangxi Medical College, Nanchang, China; ^2^ Jiangxi Hypertension Research Institute, Nanchang, China

**Keywords:** case report, coronary artery disease, diagonal earlobe crease, Frank's sign, pre-existing cardiac disease

## Abstract

Frank's sign (FS) refers to a diagonal skin fold between the tragus and the outer edge of the earlobe. FS has been identified as an independent variable in coronary artery disease (CAD). Young patients with FS and previous myocardial infarction are still rarely reported in clinical studies. We report the case of a 49-year-old male smoker and diabetic, with a history of myocardial infarction, who presented to the emergency department due to 2 h typical cardiac chest pain. His urgent electrocardiography (ECG) showed ST elevation, and cardiac biomarkers were elevated after admission. A diagonal earlobe crease (DELC) was observed in physical tests. The preliminary diagnosis considered acute coronary syndrome (ACS). Subsequently, acute coronary artery angiography demonstrated a slit-like contrast defect in the proximal right coronary artery (RCA), with stenosis and occlusion in the distal segment. The percutaneous coronary intervention (PCI) was performed immediately. The patient's chest pain symptoms were relieved significantly after intervention. Our case indicates that FS should be highly regarded as a routine cardiovascular clinical examination, which can be effortlessly applied and be easily interpreted for screening to suspect the presence of ischemic heart disease. This may set strategies for primary screening in a younger population and prompt early diagnosis and treatment.

## 1. Introduction

Frank's sign (FS), also known as the diagonal earlobe crease (DELC), refers to a diagonal skin fold between the tragus and the outer edge of the earlobe. It was first described as a predictive dermatological finding of coronary artery disease (CAD) in 1973 by the American pulmonologist Sanders T. Frank [1]. The FS was graded according to the following system: Grade 1 is a slight wrinkling of the skin around the earlobe. Grade 2a involves a superficial skin fold that partially covers the earlobe at least halfway; Grade 2b involves a superficial skin fold that covers the earlobe completely. Grade 3 involves a deep skin fold that covers the entire earlobe [2].

FS correlates with cardiovascular risk factors, especially CAD. The detailed mechanisms underlying FS remain unclear. This sign is considered a crucial diagnostic physical examination for predicting atherosclerotic disease in people younger than 60 years of age [3]. The association between FS and prognosis after acute myocardial infarction (AMI) requires further investigation [4]. Even if the clinical diagnostic accuracy of FS remains uncertain, this physical examination should serve as a meaningful warning because of its feasibility and ease of interpretation [5].

## 2. Case Presentation

**Figure 1:**
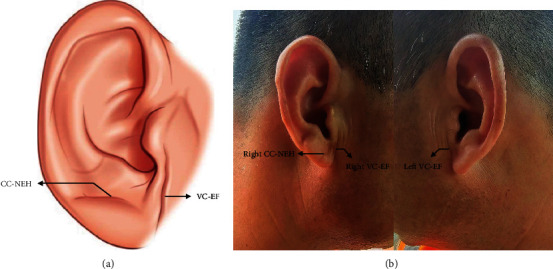
(a) Two prominent ear creases on the ears. VC-EF, vertical creases dividing earlobe and face (including right VC-EF and left VC-EF); CC-NEH, crossing creases not originated from ear hole (including right CC-NEH and left CC-NEH). (b) The right CC-NEH and bilateral VC-EF were found on our patient's ears.

**Figure 2:**
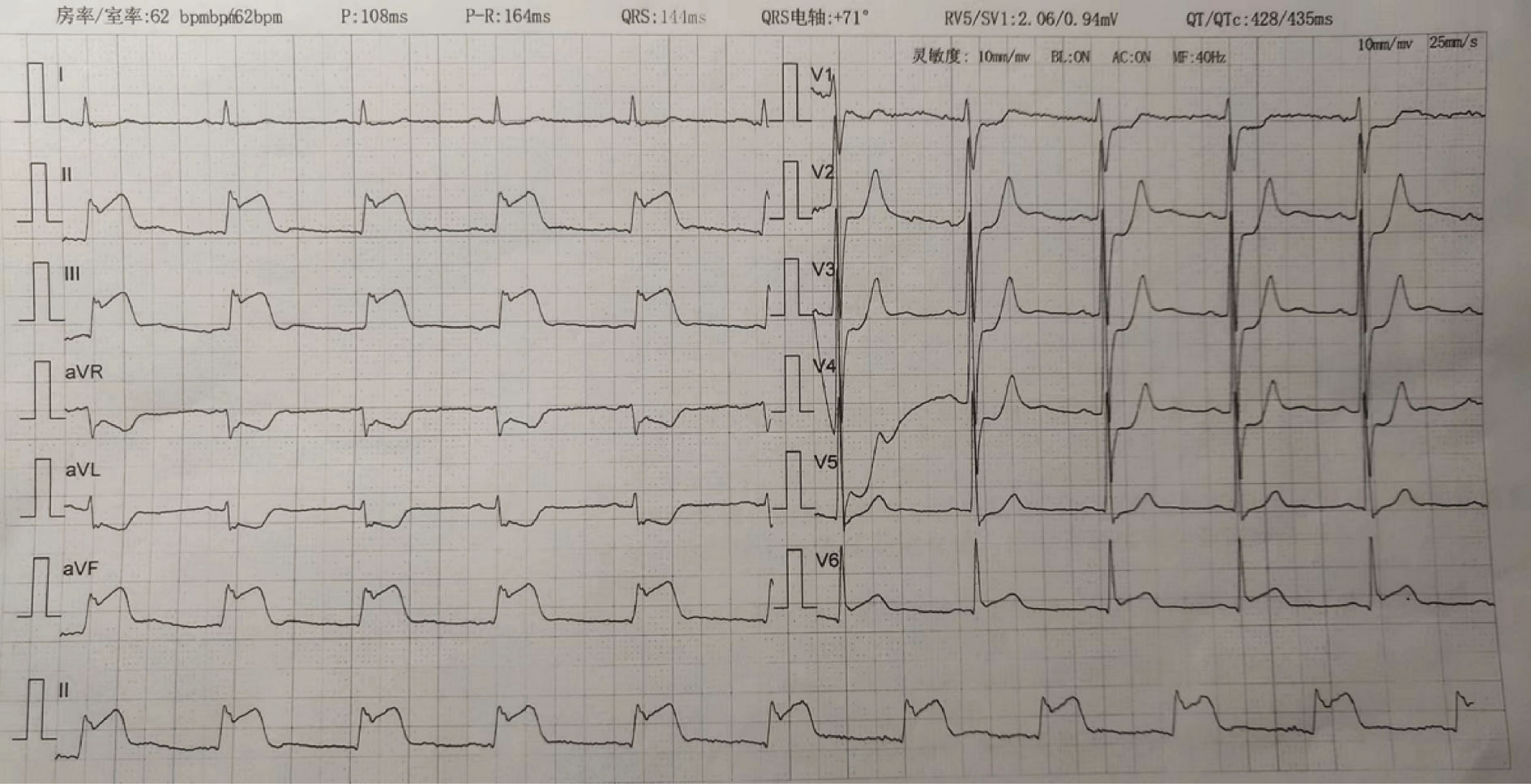
The initial electrocardiogram (ECG) revealed ST-segment elevation (STE) in leads II, III, and aVF with reciprocal depression in leads I, aVL, and V1-V4. The ratio of sum of ST-segment depression (STD) in leads V1–V3/sum of STE in leads II, III, and aVF < 1.

A 49-year-old male patient presented to our hospital's emergency department complaining of the sudden onset of chest pain lasting for 2 h with a sense of compression, about the size of the palm, and radiation to the back. Feelings of fatigue and nausea also persisted. There was no obvious remission after rest. His medical history included Type 2 diabetes mellitus, a smoking history of 20 cigarettes per day, and a previous myocardial infarction in 2021 with stent implantation in the circumflex artery. His family history included his mother's coronary heart disease. His prior medications included voglibose (0.2 mg tid), gliclazide (30 mg qd), aspirin (100 mg qd), ticagrelor (90 mg bid), and atorvastatin calcium (20 mg qn). After admission, electrocardiography (ECG) and myocardial enzyme levels were urgently examined. The vital signs of the patient's admission were temperature of 36.5°C, pulse rate of 59 beats per minute, respiration of 20 breaths per minute, blood pressure of 116/100 mmHg, and blood oxygen saturation of 100%. During the physical examination, we found the presence of FS in the patient's right ear, while the presence of vertical creases dividing earlobe and face (VC-EF) was found in both ears [6] (Figure 1). His initial ECG revealed ST-segment elevation (STE) in leads II, III, and aVF with reciprocal depression in leads I, aVL, and V1–V4. The ratio of sum of ST-segment depression (STD) in leads V1–V3/sum of STE in leads II, III, and aVF < 1 [7] supports right coronary artery (RCA) (Figure 2). Laboratory tests suggested that TnI was 0.023 *μ*g/L (RI: 0–0.023 *μ*g/L), creatine kinase (CK) was 113.1 U/L (RI: 50–310 U/L), CK isoenzyme (CK-MB) was 16.0 U/L (RI: 0–24 U/L), lactate dehydrogenase was 245.3 U/L (RI: 120–250 U/L), potassium was 3.85 mmol/L (RI: 3.5–5.3 mmol/L), and HbA1C was 6.5% (RI: 4.5%–6.3%). Furthermore, blood tests showed normal liver and renal functions. The primary diagnosis was considered acute coronary syndrome (ACS). The second day after admission, the re-examination of myocardial enzyme levels suggested that CK was 3335.0 U/L and CK-MB was 188.0 U/L. The changes in myocardial enzyme levels further confirmed our diagnosis.

**Figure 3:**
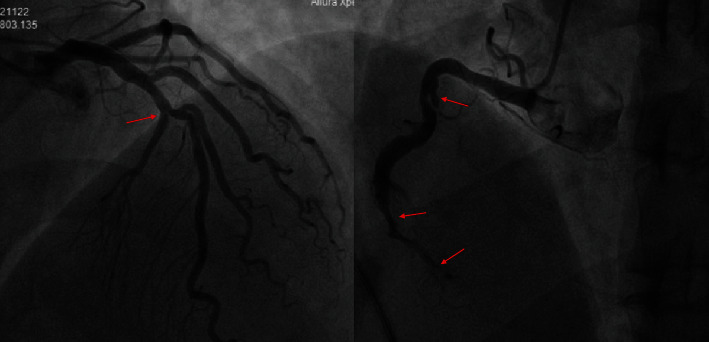
Coronary angiography (CAG) showed a slit-like contrast defect in the proximal right coronary artery (RCA), with stenosis and occlusion in the distal segment. Moreover, diffuse lesions were observed in the proximal left anterior descending (LAD) artery, with 50%–60% stenosis at the most severe site. Approximately 40%–50% stenosis in the proximal left circumflex artery (LCX) and a stent shadow in the middle LCX with no obvious stenosis in the stent can be seen (the arrows refer to coronary stenosis).

**Figure 4:**
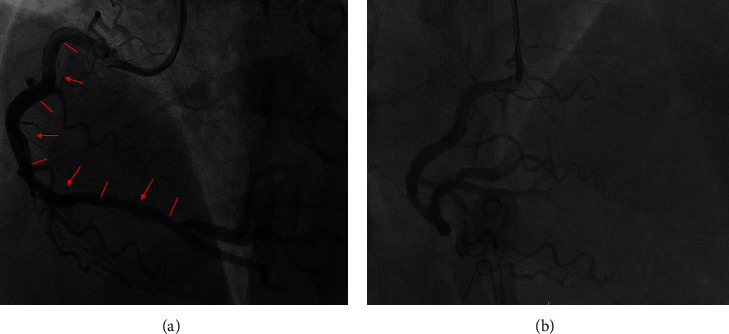
Repeat coronary angiography (CAG) after the right coronary artery percutaneous coronary intervention (PCI). Blood flow recovery at occlusion of the right coronary artery (RCA), and the thrombolysis in myocardial infarction blood flow in the distal right coronary artery was Grade 3. (a) The locations of the four delivered stents were marked in red arrows. (b) The middle segment of the RCA under RAO-CRA projection after PCI.

**Figure 5:**
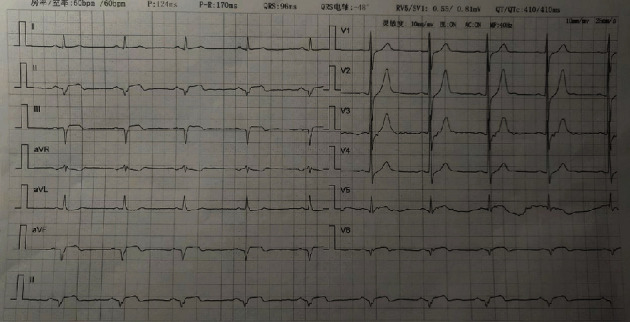
The postprocedural electrocardiogram (ECG) showed leads II, III, and aVF fell back and necrotic Q wave appeared, while leads I, aVL, and V1–V4 returned to the equipotential line.

After receiving a loading dose of aspirin 300 mg and ticagrelor 180 mg with the consent of the patient's family, he underwent urgent coronary angiography (CAG) due to persistent symptoms. CAG results showed a slit-like contrast defect in the proximal RCA, with stenosis and occlusion in the distal segment. Moreover, diffuse lesions were observed in the proximal left anterior descending (LAD) artery, with 50%–60% stenosis at the most severe site. Approximately 40%–50% stenosis in the proximal left circumflex (LCX) artery and a stent shadow in the middle LCX with no obvious stenosis in the stent can be seen (Figure 3). After consulting the patient's family, we subsequently performed percutaneous coronary intervention (PCI) of the RCA. Four drug-eluting stents (2.75 × 36 mm, 3.0 × 38 mm, 3.5 × 26 mm, and 3.5 × 26 mm) were successfully placed in the RCA. Repeat angiography showed blood flow recovery at occlusion of the RCA, and the thrombolysis in myocardial infarction blood flow in the distal RCA was Grade 3(Figure 4). After the intervention, the patient's chest pain symptoms improved significantly, and he returned to the coronary care unit. The postprocedural ECG showed that leads II, III, and aVF fell back and necrotic Q wave appeared, while leads I, aVL, and V1–V4 returned to the equipotential line (Figure 5). The patient's left ventricular ejection fraction on the first postoperative day was 55%. No complications occurred during the hospitalization. The patient was discharged 6 days after PCI. The patient was followed up in the outpatient clinic of our hospital every 3 months after discharge. During the 1-year follow-up, he did not complain of any chest pain symptoms.

## 3. Discussion

Ischemic heart disease is the leading cause of cardiovascular death worldwide, especially in patients with STE myocardial infarction (STEMI). Physical examination remains a significant diagnostic indicator of cardiovascular disease. Some visible age-related signs, such as FS, male pattern baldness, and xanthelasmata, are often associated with an increased risk of ischemic heart disease [3]. FS, viewed as an outcome correlated with an increase in facial visceral fat deposition and the persistence of auricular traction, is often a dermatological indicator of cardiovascular disease [8].

A lot of studies have reported that FS is a useful cutaneous physical finding in patients with atherosclerotic diseases. The pathophysiology of this mechanism is not yet clear. Several hypotheses have been put forth to explain the phenomenon, such as excessive telomere loss and an external sign of a microangiopathic lesion of terminal vessels. A study revealed that the serum levels of inflammatory biomarkers (high-sensitivity C-reactive protein, pentraxin 3) and oxidative stress markers (malondialdehyde low-density lipoprotein) were found to be significantly higher in the FS groups than in the non-FS groups [9]. Furthermore, serum pentraxin 3 was considered the strongest factor for predicting the prevalence of FS [9]. Enhanced oxidative stress and inflammation could be common pathways linking FS and CAD [9]. Low adropin and irisin were recently considered a plausible common pathological basis for atherosclerosis and FS [10]. Endothelial dysfunction due to adropin and irisin deficiencies may be a bridge connecting CAD and FS, which explains why patients with FS are prone to CAD [10]. It remains to be further confirmed whether adropin and irisin can be regarded as future severe heart disease predictors in patients with FS.

Studies have shown that the presence of FS is closely correlated with the complexity of coronary lesions [6]. A FS score was established based on the intermediate and high SYNTAX score (score ≥ 23)-associated ear creases-VC-EF and crossing creases not originated from ear hole (CC-NEH) [6]. Each ear crease of left VC-EF, right VC-EF, left CC-NEH, and right CC-NEH was scored as 1 point; then, the patients could have 0–4 points according to the ear creases, with the score constituting the FS scores [6]. The FS score = 0 may be used as the exclusion criteria for intermediate and high SYNTAX score, while the FS scores = 3 or 4 should receive much more attention since up to 50% of these patients have very complicated and potentially lethal coronary lesions [6]. FS was independently and significantly associated with extent and severity of CAD in a study consisting of 356 patients with first diagnosis of ACS [11]. The coronary atherosclerosis burden was more advanced in patients with FS detected on the physical examination at first presentation of ACS [11]. In addition, it was reported that the FS was a statistically significant predictor of a residual SYNTAX score of ≥9 in STEMI patients, which might mean poor long-term prognoses [12]. Another cross-sectional study aimed at patients ≤ 65 years old with FS revealed that multivessel disease and/or chronic total occlusion was found in patients from 45 years old [13].

FS was thought to be connected with a long-term prognosis. The occurrence of FS was reported to be more common in the presence of cardiovascular risk factors such as hypertension and diabetes and more frequently in the presence of a previous myocardial infarction or ischemic heart disease [2]. Furthermore, the high-grade FS had a higher grade of CAD severity and atherosclerosis. The FS showed a higher sensitivity but a lower specificity for the diagnosis of a pre-existing cardiac disease compared to age, and the FS seemed to be of prognostic value for younger patients [2]. A prospective examination revealed that the association between FS and mortality was considerably strong in the first year after AMI, and it was also statistically significant [4]. In the fully adjusted Cox regression model, patients with FS Grade 2/3 had a 2.57-fold increased risk of death compared to the patients with FS Grade 0/1. (CI 1.07–6.17, *p* = 0.0347) [4].

Our patient was a young man with Type 2 diabetes mellitus and a previous myocardial infarction. His unilateral FS was considered as Grade 2a involved a superficial skin fold that partially covered the earlobe at least halfway. His FS scores were 3 (including left VC-EF, right VC-EF, and right CC-NEH). Severe CAD was found in the CAG. His SYNTAX scores were about 28.5 (score ≥ 23), and his residual SYNTAX scores were about 9 (score ≥ 9). Nevertheless, the patient did not suffer from serious adverse cardiovascular events during outpatient follow-up within 1 year.

In addition, we found a slit contrast defect in the proximal RCA. We thought this was more likely to be considered a niche formed by coronary ulcer rather than a dissection. It usually represents the unstable state of atherosclerotic plaque, which can easily induce the formation of local thrombus. Noniatrogenic dissection is more spontaneous coronary artery dissection, which is more likely to occur in women around 50 years who have no risk factors for atherosclerosis [14]. Coronary ulcers also need to be identified with coronary aneurysms in clinic.

Attention to this sign enabled us to carry out treatment work more quickly. We think it is useful to have an indicator such as an easily detectable physical sign to immediately recognize STEMI with poor prognoses in acute settings, especially to patients who are not known to be at risk for atherosclerosis or whose condition is too critical to be interviewed for their medical history. Furthermore, application of “Frank's sign score,” which can be helpful for screening and primary prevention, may make the evaluation of coronary lesions via ear creases easy and precise. The early warning role of FS signs in critical patients with suspected AMI is worthy of further study. The prognostic effect of residual SYNTAX score in STEMI patients with FS deserves further study. Further improvement in follow-up is necessary. The main limitation of this paper is that the intravascular ultrasound technique is not used in the process of intervention.

## 4. Conclusions

Our patient exhibited FS scores = 3 and had a history of myocardial infarction. The patient was admitted due to total occlusion of the RCA. FS is useful for early detection in young patients with a high probability of presenting severe CAD. The intentional physical examination for FS in a first contact clinic scenario can be effortlessly applied, and the application of the FS score may provide a quick, easy, and effective assessment of complicated CAD. This may set strategies for primary screening in a younger population and prompt early diagnosis and treatment.

## Data Availability

Data supporting this research article are available from the corresponding author or first author on reasonable request.

## Nomenclature

ACS: acute coronary syndrome
AMI: acute myocardial infarction
CAD: coronary artery disease
CAG: coronary angiography
CC-NEH: crossing creases not originated from ear hole
CK: creatine kinase
CK-MB: creatine kinase isoenzyme
DELC: diagonal earlobe crease
ECG: electrocardiography
FS: Frank's sign
LAD: left anterior descending
LCX: left circumflex
PCI: percutaneous coronary intervention
RCA: right coronary artery
STD: ST-segment depression
STE: ST-segment elevation
STEMI: ST-segment elevation myocardial infarction
VC-EF: vertical creases dividing earlobe and face

## References

[1] Frank S. T. (1973). Aural sign of coronary-artery disease. *The New England Journal of Medicine*.

[2] Prangenberg J., Doberentz E., Johann L., Madea B. (2022). The prognostic value of the Frank sign. *Forensic Science, Medicine, and Pathology*.

[3] Ono R., Iwahana T., Kobayashi Y. (2020). Frank's sign in recurrent triple-vessel disease. *BMJ Case Reports*.

[4] Thilo C., Meisinger C., Heier M., von Scheidt W., Kirchberger I. (2021). Diagonal earlobe crease and long-term survival after myocardial infarction. *BMC Cardiovascular Disorders*.

[5] Więckowski K., Gallina T., Surdacki A., Chyrchel B. (2021). Diagonal earlobe crease (Frank's sign) for diagnosis of coronary artery disease: a systematic review of diagnostic test accuracy studies. *Journal of Clinical Medicine*.

[6] Liu Z., Qiu C., Xu J. (2020). Ear crease features are associated with complexity of coronary lesions. *Medical Science Monitor*.

[7] Fiol M., Carrillo A. (2010). Culprit artery in evolving inferior wall acute myocardial infarction: RCA vs LCX. *Europace*.

[8] Abrahim M. (2022). Unified anatomical explanation of diagonal earlobe creases, preauricular creases, and paired creases of the helix. *Cureus*.

[9] Koyama T., Watanabe H., Ito H. (2016). The association of circulating inflammatory and oxidative stress biomarker levels with diagonal earlobe crease in patients with atherosclerotic diseases. *Journal of Cardiology*.

[10] Wei N., Zhang R., Zhu Z. (2021). Adropin and irisin deficiencies are associated with presence of diagonal earlobe crease in cad patients. *Frontiers in Cardiovascular Medicine*.

[11] Gayretli Yayla K., Özbay M., Yakut I. (2022). Frank sign may predict more advanced coronary artery disease in patients admitted with a first time acute coronary syndrome. *Turkish Journal of Clinics and Laboratory*.

[12] Kaichi R., Kawakami S., Tahara Y. (2024). Relationship between earlobe crease and anatomical severity of coronary artery disease in ST-segment elevation myocardial infarction. *Internal Medicine*.

[13] Velázquez-Sotelo C. E., Fernández-Gómez M. J., Cázares-Pérez A. (2023). Frank's sign associated with the severity of ischemic heart disease in patients under 65 years old. *Medicina Clinica*.

[14] Kim E. S. H. (2020). Spontaneous coronary-artery dissection. *The New England Journal of Medicine*.

